# Risk factors for unanticipated hypertension during emergence from general anesthesia in elderly surgical patients: A retrospective cohort analysis

**DOI:** 10.1097/MD.0000000000049318

**Published:** 2026-06-26

**Authors:** Shuang Shuang Jiao, Xing Jie Guo, Li Xiang Yu

**Affiliations:** a Department of Anesthesiology, Nanjing BenQ Medical Center, The Affiliated BenQ Hospital of Nanjing Medical University, Nanjing, Jiangsu, China.

**Keywords:** elderly patients, general anesthesia, postanesthesia care unit (PACU), risk factors, unanticipated hypertension

## Abstract

Unanticipated hypertension in the postanesthesia care unit (PACU) refers to acute blood pressure elevation in patients whose preoperative baseline blood pressure was normal, possibly resulting in adverse postoperative complications. This study aimed to identify potential factors and the impact of unanticipated hypertension during emergence from general anesthesia in the elderly surgical population. A retrospective study was done at the Affiliated BenQ Hospital of Nanjing Medical University. Elderly surgical patients who had been admitted to the PACU for recovery from general anesthesia were enrolled. The demographic and clinical variables of elderly patients were systematically analyzed. All elderly patients were divided into the case group and the control group based on whether they experienced unanticipated hypertension during the anesthesia recovery period. Conditional multivariate logistic regression was performed to investigate factors responsible for unanticipated hypertension during the general anesthesia recovery period. Among the 840 enrolled elderly patients, 212 (25.2%) developed unanticipated hypertension during emergence from general anesthesia. Multivariate logistic regression analyses, advanced age (odds ratio [OR] = 2.016, 95% confidence interval [CI]: 1.458–2.824, *P* < .001), female sex (OR = 1.494, 95% CI: 1.049–2.130, *P* = .026), absence of antihypertensive medication use (OR = 1.886, 95% CI: 1.319–2.717, *P* = .001), orthopedic surgery (OR = 2.006, 95% CI: 1.302–3.082, *P* = .002), intraoperative hypertension (OR = 1.587, 95% CI: 1.104–2.279, *P* = .012), urinary catheterization (OR = 3.674, 95% CI: 2.222–6.350, *P* < .001), absence of patient-controlled analgesia (OR = 2.774, 95% CI: 1.887–4.094, *P* < .001), postoperative pain (OR = 5.692, 95% CI: 3.003–11.006, *P* < .001), and postoperative agitation (OR = 1.878, 95% CI: 1.263–2.784, *P* = .002) were independently associated with unanticipated hypertension during anesthetic emergence in geriatric surgical patients. Furthermore, those who experienced unanticipated hypertension exhibited significantly higher rates of delayed extubation and prolonged stays in the PACU. Advanced age, female sex, absence of preoperative antihypertensive medication, orthopedic surgery, intraoperative hypertension, urinary catheterization, postoperative pain, and postoperative agitation were independently associated with an increased risk of unanticipated hypertension during emergence from anesthesia in elderly surgical patients. These findings underscore the importance of proactive and well-structured postoperative blood pressure management strategies in clinical anesthetic practice.

## 1. Introduction

Approximately 40 million noncardiac surgical procedures are performed annually in patients aged 65 years or older worldwide, accounting for over 30% of all major surgeries globally. Contemporary evidence indicates that 15% to 30% of geriatric patients experience significant postoperative complications within 30 days following noncardiac surgery.^[1]^ Notably, 40% to 60% of these complications manifest during the critical perioperative phase in the postanesthesia care unit (PACU), where acute postoperative hemodynamic instability accounts for 18% to 22% of all adverse events.^[2]^ One such physiological disturbance is characterized by elevated blood pressure, with an incidence of 7.8 cases per 100 admissions.^[3]^ Unstable hemodynamic alterations may significantly exacerbate the physiological reserve burden in elderly patients during the postoperative period. Unanticipated hypertension in the PACU is defined as an acute elevation in blood pressure following general anesthesia in patients whose preoperative baseline blood pressure was normal.^[4]^ Despite its clinical significance, this condition has historically received limited attention due to the assumption that patients with normotensive preoperative profiles are at low risk. However, emerging evidence indicates that untreated acute postoperative hypertension may precipitate severe postoperative complications, including rupture of vascular anastomoses^[5]^ and cardiovascular or cerebrovascular events.^[6,7]^ These adverse outcomes, which remain prevalent after noncardiac surgery, are associated with prolonged intensive care unit stays and increased postoperative mortality.^[8,9]^

Since 1975, previous studies^[10–12]^ have reported risk factors for acute postoperative hypertension, primarily influenced by patient-specific, anesthetic, and surgical variables. Given that elderly patients are predisposed to essential hypertension and advanced age has been consistently established as an independent risk factor for acute postoperative hypertension, this population warrants heightened vigilance from anesthesiologists. However, existing studies have been constrained by small sample sizes and a narrow focus on single surgical procedures, limiting the generalizability and robustness of their findings. Unanticipated hypertension following general anesthesia has a higher likelihood of resulting in many adverse postoperative outcomes, which is partly explained by its unintended nature. This risk is amplified by the fact that such hypertensive episodes are often unpredictable and may occur during critical transitional phases, such as emergence from anesthesia. Early identification of high-risk elderly individuals may facilitate more informed perioperative management decisions and ultimately improve postoperative prognosis.^[13]^ We therefore aimed to identify predictors of unanticipated hypertension in elderly surgical patients during emergence from anesthesia by leveraging extensive real-world clinical data collected from the anesthesia center at a large tertiary hospital, with the goal of informing targeted clinical interventions.

## 2. Methods

### 2.1. Ethics approval

This study protocol was reviewed and approved by the Ethics Committee of the Affiliated BenQ Hospital of Nanjing Medical University (2025-KL072). As this research involves minimal risk to participants and consists of a retrospective analysis of routinely collected clinical data, the committee determined, following comprehensive review, that the requirement for written informed consent could be waived. Prior to initiating data collection, the research team prospectively defined the primary outcome measures, data extraction procedures, and statistical analysis plan in accordance with the prespecified study protocol.

### 2.2. Study design and participants

This study employed a retrospective cohort design to investigate elderly patients who underwent noncardiac surgical procedures at our institution between March 2024 and October 2025. All included participants received general anesthesia and were transferred to the PACU for standardized postoperative monitoring and recovery. Regarding patient transfer, all patients remained under general anesthesia with an endotracheal tube or a laryngeal mask airway during transport from the operating room to the PACU. This standardized transfer protocol was applied to all patients to minimize variations in sympathetic stimulation during transport. The inclusion criteria were as follows: elective noncardiac surgery performed under general anesthesia; admission to the PACU for structured monitoring and resuscitation; and normal preoperative baseline blood pressure. Patients were excluded if they met any of the following conditions: absence of blood pressure measurements during the PACU stay; severe hepatic or renal dysfunction; or significant cardiac dysfunction.

All elderly surgical patients were categorized into an unanticipated hypertension group (case group) and a control group based on the occurrence of unanticipated hypertension during emergence from general anesthesia. The primary outcome was diagnosed using the following criteria: preoperative blood pressure within the normal range upon admission, and an increase of at least 30% in the highest recorded systolic (SBP) or diastolic blood pressure relative to this baseline value.^[4]^ In this study, delayed postoperative extubation was defined as endotracheal tube removal occurring more than 60 minutes after arrival in the PACU.^[14]^

### 2.3. Data collection

All patient data were retrieved from the Electronic Medical Records and Surgical Anesthesia Information System at our institution, which has systematically archived perioperative information since 2015. The variables included in this study encompassed baseline demographic characteristics, preoperative clinical parameters, anesthesia-related details, surgical factors, and postoperative data collected during the PACU stay. We specifically examined preexisting comorbidities previously shown to be associated with unanticipated hypertension, including essential hypertension, diabetes mellitus, history of stroke, coronary heart disease, meningioma, and anemia. Additionally, information on preoperative antihypertensive medication use among elderly surgical patients was collected. Anesthetic and surgical variables – such as surgical type, operative duration, intraoperative invasive arterial pressure monitoring, administration of anesthetic agents, intravenous fluid management, and the use of epidural catheters or patient-controlled analgesia pumps – were all documented in the case report form.

Baseline blood pressure was defined as the blood pressure measured in the ward under quiet conditions on the day before surgery. Patients with a history of essential hypertension were eligible for inclusion if their in-ward baseline blood pressure was within the normal range, reflecting adequately controlled blood pressure prior to surgery. Noninvasive arterial blood pressure measurements, including SBP and diastolic blood pressure, were recorded at 3-minute intervals immediately upon admission to the PACU during the postoperative period. In addition, this study captured key indicators of postoperative emergence quality in elderly patients, such as extubation time and time to recovery. Data on the administration of postoperative medications – specifically analgesics and supplemental propofol – were also collected to reflect clinical responses to postoperative pain and agitation.

### 2.4. Data management and statistical analysis

According to recommendations from a retrospective study, the number of outcome events in a logistic regression analysis should be at least 15 to 20 times the number of independent variables.^[15]^ In the preliminary phase of this study (n = 200), 14 potential predictors were initially entered into a multivariate logistic regression model. The observed incidence of unanticipated hypertension in elderly patients upon emergence from anesthesia was 25.6%. Taking this incidence rate into account and allowing for an estimated 2% dropout or exclusion rate, the required total sample size was determined to be 836 participants to ensure adequate statistical power.

A key methodological strength of this study is the implementation of multiple imputation to address missing data, thereby effectively reducing potential bias in variable estimation. Descriptive statistics were used to summarize patient characteristics. Continuous variables were presented as mean ± standard deviation for normally distributed data or median (interquartile range) for skewed distributions, based on distributional properties. Categorical variables were reported as frequency (percentage). Group comparisons were performed using independent samples *t* tests for normally distributed continuous variables and Mann–Whitney *U* tests for non-normally distributed data, as appropriate. For categorical outcomes, χ^2^ tests or the Fisher exact test were applied according to the expected cell frequencies to ensure statistical validity.

The association between clinical factors and the outcome variable was evaluated through univariate logistic regression analysis. To identify independent predictors, a multivariate logistic regression model was constructed, including variables that were statistically significant (*P* < .05) in the univariate analysis and those considered clinically relevant. Multicollinearity was assessed by calculating the variance inflation factor. For each covariate in the final model, both crude odds ratios (ORs) and adjusted ORs, along with their corresponding 95% confidence intervals (CIs), were estimated.

Model performance was assessed using the receiver operating characteristic (ROC) curve and calibration plots. The ROC curve was utilized to evaluate the discriminative capacity of the final model, whereas the Hosmer–Lemeshow test was applied to examine model calibration and overall goodness of fit.^[16]^ All statistical analyses were performed with R software (version 4.1.2; The R Foundation for Statistical Computing). A two-sided α level of 0.05 was used to define statistical significance.

## 3. Results

### 3.1. Demographic and perioperative clinical characteristics

**Figure 1.**
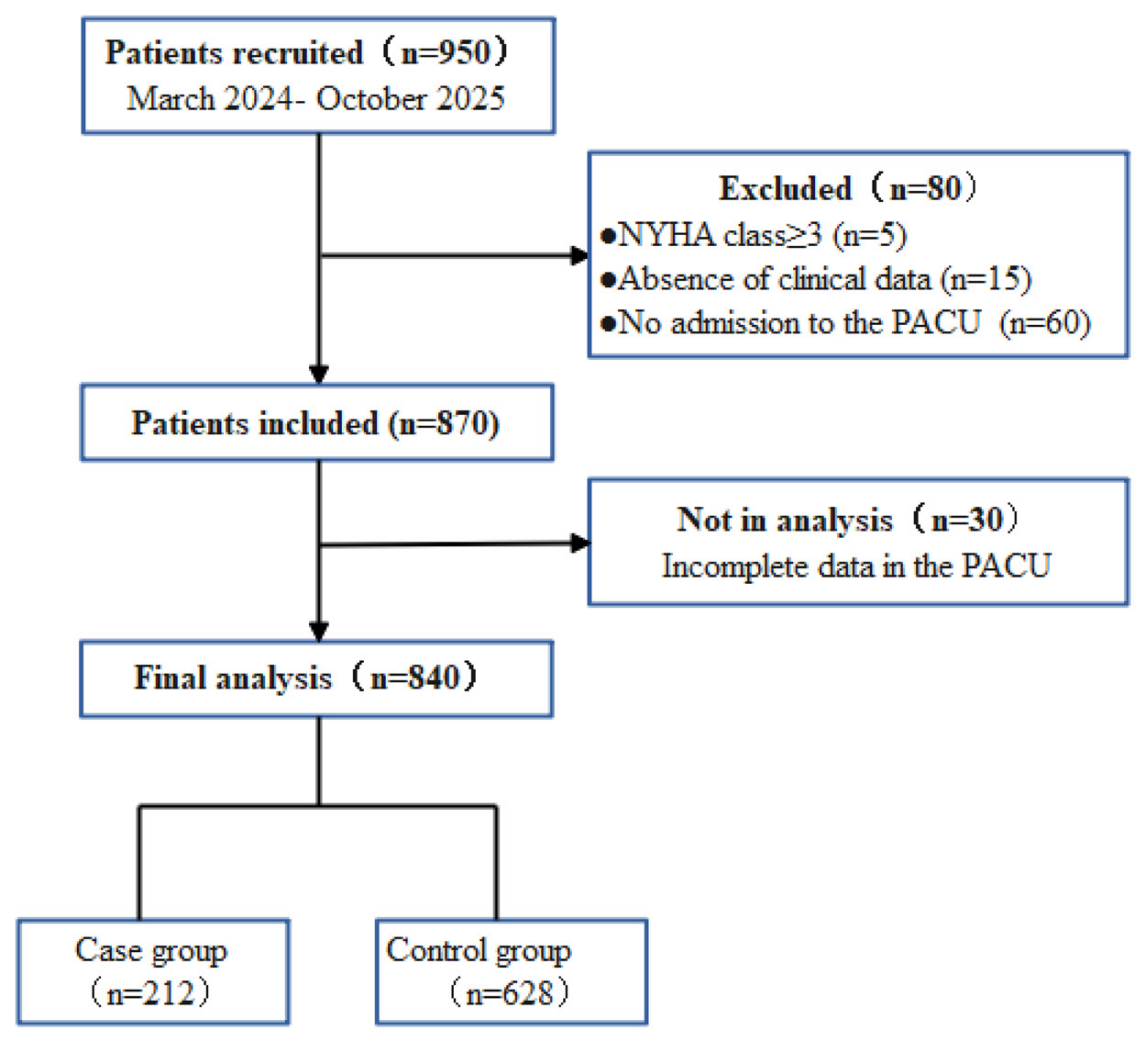
Flowchart of patient data. NYHA = New York Heart Association, PACU = postanesthesia care unit.

A total of 950 elderly patients underwent general anesthesia between March 2024 and October 2025. After applying eligibility criteria, 840 patients were included in the final analysis (Fig. 1). Among these surgical patients, 212 (25.2%) experienced unanticipated hypertension during emergence from anesthesia. The mean age of the study population was 71 years, and approximately 43.3% were female (Table 1).

**Table 1:** Patient factors associated with unanticipated hypertension.

Variable	Overall	Case group	Control group	*P*-value
No. of patients	840	212	628	
Gender				.015
Male	476 (56.7)	105 (49.5)	371 (59.1)	
Female	364 (43.3)	107 (50.5)	257 (40.9)	
Age (yr)	70.83 ± 5.76	72.49 ± 7.93	70.27 ± 4.69	<.001
BMI (kg/m^2^)*	23.29 ± 3.359	22.99 ± 3.29	23.40 ± 3.37	.127
Preoperative comorbidities				
Diabetes	151 (18.0)	29 (13.7)	122 (19.4)	.060
Hypertension	379 (45.1)	93 (43.9)	286 (45.5)	.672
Stroke	102 (12.1)	32 (15.1)	70 (11.1)	.128
Coronary heart disease	60 (7.1)	11 (5.2)	49 (7.8)	.201
Meningioma	24 (2.9)	4 (1.9)	20 (3.2)	.327
Anemia	204 (24.3)	64 (30.2)	140 (22.3)	.020
No_hypotensive drugs	477 (56.8)	143 (67.5)	334 (53.2)	<.001
ASA classification				.007
I	7 (0.8)	0 (0.0)	7 (1.1)	
II	672 (80.0)	157 (74.1)	515 (82.0)	
III	158 (18.8)	53 (25.0)	105 (16.7)	
IV	3 (0.4)	2 (0.9)	1 (0.2)	

Data were presented as count (percentage) or mean ± SD.

ASA = American Society of Anesthesiologists Physical Status Classification, BMI = body mass index, SD = standard deviation.

* Missing data.

#### 3.1.1. Patient-related factors

The majority of patients (80.0%) were classified as American Society of Anesthesiologists Physical Status Classification II. Regarding comorbidities among elderly patients, 45.1%, 18.0%, 12.1%, and 7.1% had essential hypertension, diabetes, stroke, and coronary heart disease, respectively (Table 1). The prevalence of primary hypertension did not differ significantly between the 2 groups. Notably, 56.8% of patients diagnosed with primary hypertension had not adhered to prescribed preoperative antihypertensive medications.

#### 3.1.2. Anesthetic- and surgical-related factors

Intraoperative hypertension was observed in 281 elderly patients (33.5%), and approximately 12.4% of these patients received intraoperative blood transfusions. In this study, the majority of elderly patients (77.3%) had an indwelling catheter placed during surgery, which was maintained into the postoperative period (Table 2). Additionally, 791 (94.2%) elderly patients did not transition to a patient-controlled analgesic pump in a timely manner at the conclusion of surgery.

**Table 2:** Anesthesia and surgical factors associated with unanticipated hypertension.

Variable	Overall	Case group	Control group	*P*-value
No. of patients	840	212	628	
Urgency of surgery				
Elective	804 (95.7)	201 (94.8)	603 (96.0)	.453
Emergency	36 (4.3)	11 (5.2)	25 (4.0)	
Anesthesia time (min)	178.5 ± 90.25	174.2 ± 93.65	179.9 ± 89.10	.423
Operation time (min)	152.1 ± 86.88	147.6 ± 90.50	153.6 ± 85.64	.383
Operation time >3 h	264 (31.4)	55 (25.9)	209 (33.3)	.047
Orthopedic surgery	166 (19.8)	54 (25.5)	112 (17.8)	.016
Intraoperative hypertension				.018
Yes	281 (33.5)	85 (40.1)	196 (31.2)	
No	559 (66.5)	127 (59.9)	432 (68.8)	
Intubation method				.859
Endotracheal tube	702 (83.6)	178 (84.0)	524 (83.4)	
Laryngeal mask	138 (16.4)	34 (16.0)	104 (16.6)	
Anesthetic agents				.709
Sufentanil (µg)	39.2 ± 10.82	38.9 ± 10.78	39.3 ± 10.84	
Dexmedetomidine				0.101
Yes	313 (37.3)	69 (32.5)	244 (38.9)	
No	527 (62.7)	143 (67.5)	384 (61.1)	
Total liquid (mL)	1400 ± 730.2	1345 ± 766.4	1418 ± 717.4	.203
Blood transfusion				.856
Yes	104 (12.4)	27 (12.7)	77 (12.3)	
No	736 (87.6)	185 (87.3)	551 (87.7)	
Urinary catheter				<.001
Yes	649 (77.3)	189 (89.2)	460 (73.2)	
No	191 (22.7)	23 (10.8)	168 (26.8)	
No_PCIA				<.001
Yes	791 (94.2)	183 (86.3)	608 (96.8)	
No	49 (5.8)	29 (13.7)	20 (3.2)	

Data were presented as n (%) or mean ± SD.

PCIA = patient-controlled intravenous analgesia, SD = standard deviation.

#### 3.1.3. Postoperative factors

The results of our study indicated that in the PACU, 49 elderly patients (5.8%) experienced mild to moderate pain, while emergence agitation (EA) was observed in 22.0% of these patients (Table 3). These findings are likely attributable to the administration of additional analgesics and propofol during PACU management.

**Table 3:** Postoperative factors associated with unanticipated hypertension.

Variable	Overall	Case group	Control group	*P*-value
No. of patients	840	212	628	
Postoperative extubation				.567
Yes	806 (96.0)	202 (95.3)	604 (96.2)	
No	34 (4.0)	10 (4.7)	24 (3.8)	
Supplemental analgesics				<.001
Yes	49 (5.8)	29 (13.7)	20 (3.2)	
No	791 (94.2)	183 (86.3)	608 (96.8)	
Supplemental propofol				<.001
Yes	185 (22.0)	68 (32.1)	117 (18.6)	
No	655 (78.0)	144 (67.9)	511 (81.4)	

Data were presented as n (%).

### 3.2. Factors responsible for unanticipated hypertension in the PACU

**Figure 2.**
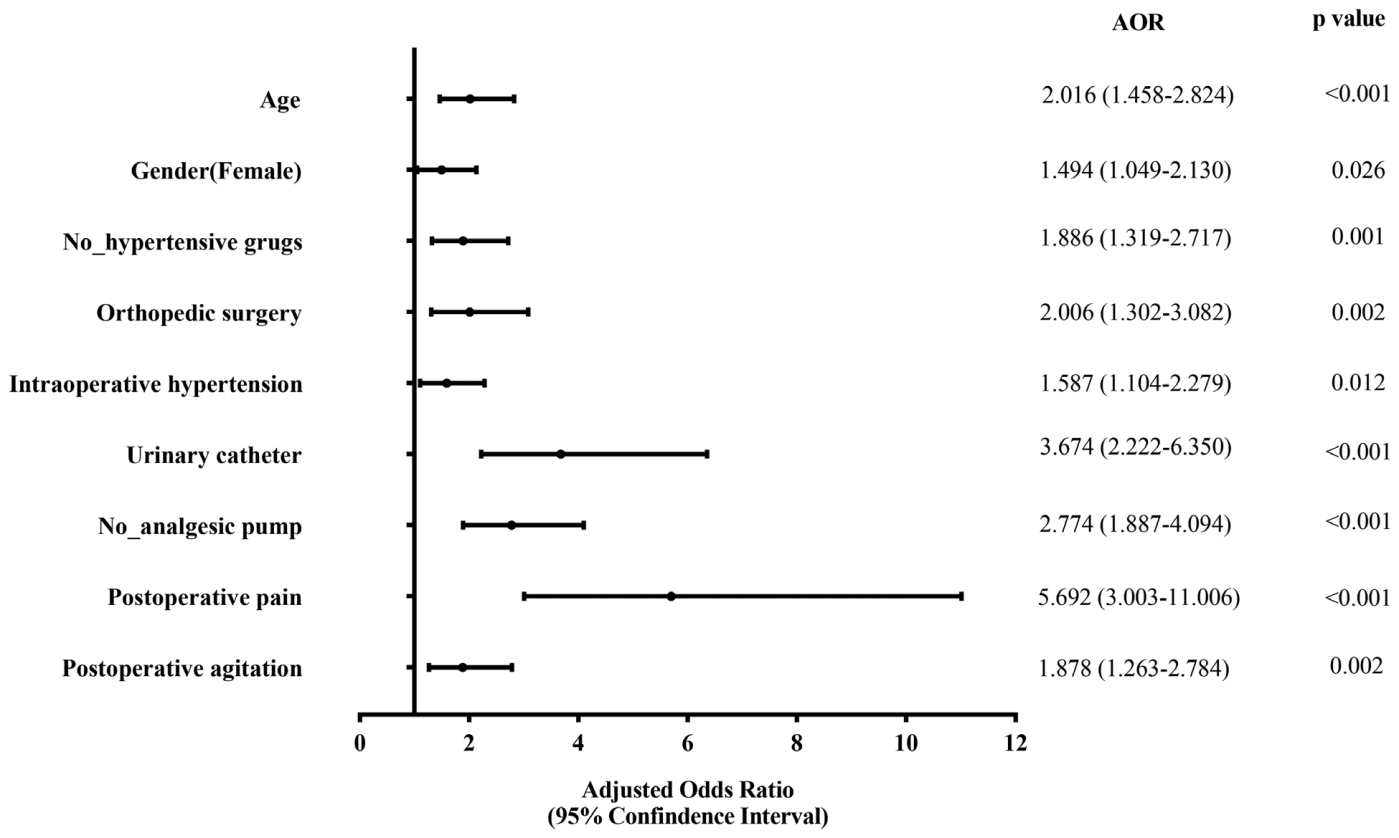
Risk factors and adjusted odds ratios (95% CI) for unanticipated hypertension during emergence from anesthesia in elderly patients. AOR = adjusted odds ratio, CI = confidence interval.

A backward stepwise multivariate logistic regression analysis was subsequently conducted to develop the final conditional model, and 9 factors were identified as independent risk factors (Table 4). After adjusting for potential confounders, the final multivariate logistic regression model demonstrated that, among elderly surgical patients, advanced age (OR = 2.016, 95% CI: 1.458–2.824, *P* < .001), female sex (OR = 1.494, 95% CI: 1.049–2.130, *P* = .026), absence of antihypertensive medication use (OR = 1.886, 95% CI: 1.319–2.717, *P* = .001), orthopedic surgery (OR = 2.006, 95% CI: 1.302–3.082, *P* = .002), intraoperative hypertension (OR = 1.587, 95% CI: 1.104–2.279, *P* = .012), urinary catheterization (OR = 3.674, 95% CI: 2.222–6.350, *P* < .001), absence of patient-controlled analgesia (OR = 2.774, 95% CI: 1.887–4.094, *P* < .001), postoperative pain (OR = 5.692, 95% CI: 3.003–11.006, *P* < .001), and postoperative agitation (OR = 1.878, 95% CI: 1.263–2.784, *P* = .002) were independently associated with unanticipated hypertension during emergence from anesthesia. The final predictive model is presented as a forest plot illustrating the individual strength of each factor, as shown in Figure 2.

**Table 4:** Logistic regression analysis for predictive factors.

Variable	Univariate logistic regression		Multivariate logistic regression	
	COR (95% CI)	*P*-value	AOR (95% CI)	*P*-value
Age/10	1.964 (1.473–2.634)	<.001*	2.016 (1.458–2.824)	**<.001**
Gender (female)	1.471 (1.076–2.012)	.016	1.494 (1.049–2.130)	**.026**
ASA classification				
I–II	Reference	–	Reference	**–**
III–IV	1.725 (1.185–2.493)	.004*	1.234 (0.788–1.913)	.352
No_ hypotensive drugs	1.925 (1.220–2.873)	<.001*	1.886 (1.319–2.717)	**.001**
Diabetes	0.657 (0.417–1.006)	.061	0.733 (0.436–1.217)	.158
Anemia	1.507 (1.060–2.130)	.021	1.328 (0.864–2.219)	.058
Orthopedic surgery	1.575 (1.082–2.273)	.016	2.006 (1.302–3.082)	**.002**
Operation time >3 h	0.702 (0.492–0.991)	.047	0.865 (0.467–1.016)	.068
Intraoperative hypertension	1.475 (1.067–2.034)	.018	1.587 (1.104–2.279)	**.012**
Urinary catheter	3.001 (1.916–4.900)	<.001*	3.674 (2.222–6.350)	**<.001**
No_analgesic pump	2.730 (1.552–3.959)	<.001*	2.774 (1.887–4.094)	**<.001**
Postoperative pain	4.817 (2.678–8.832)	<.001*	5.692 (3.003–11.006)	**<.001**
Postoperative agitation	2.062 (1.448–2.926)	<.001*	1.878 (1.263–2.784)	**.002**

The bold values are the adjusted odds ratio (AOR) and corresponding *P*-values from multivariate logistic regression analysis that reached statistical significance (*P* < .05). These values indicate independent risk factors significantly associated with unanticipated hypertension during anesthesia emergence after adjusting for confounding variables.

AOR = adjusted odds ratio, ASA = American Society of Anesthesiologists Physical Status Classification, CI = confidence interval, COR = crude odds ratio.

* Variables with *P* < .01 in univariate logistic regression analysis were statistically significant and associated with unanticipated hypertension during anesthesia emergence.

**Figure 3.**
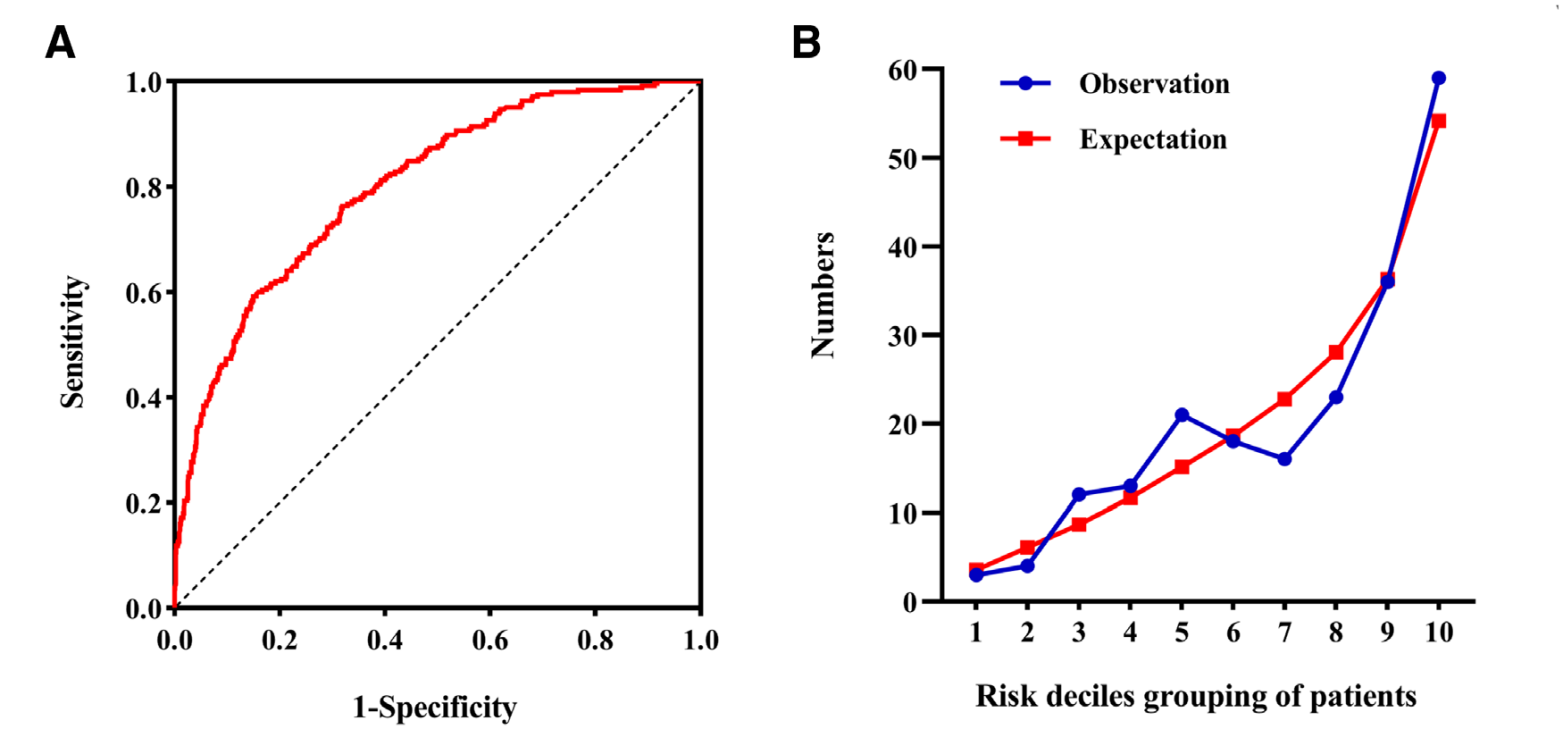
Evaluation of a multivariate logistic regression model for unanticipated hypertension during emergence from anesthesia in elderly patients: the receiver operating characteristic curve (A) and the Hosmer–Lemeshow test calibration curve (B). AUC = 0.788 (95% CI: 0.756–0.819); the calibration curve (χ^2^ = 8.110, *P* > .05). AUC = area under the ROC curve, CI = confidence interval.

The ROC curve and calibration plot are presented in Figure 3A and B. The area under the ROC curve was 0.788 (95% CI: 0.756–0.819), indicating that the model has good discriminatory accuracy for predicting unanticipated hypertension during emergence from anesthesia in elderly patients. Furthermore, the calibration curve (χ^2^ = 8.110, *P* > .05) demonstrated that the predictive values of the model were in close agreement with the observed outcomes.

### 3.3. Postoperative recovery indicators of patients in the PACU

Detailed data on exploratory short-term outcomes are summarized in Table 5. Elderly patients who experienced unanticipated hypertension had a significantly prolonged stay in the PACU, with a mean duration of 117 minutes, compared to 108 minutes for those without such events, indicating impaired early recovery. Moreover, unanticipated hypertension was also significantly associated with an increased risk of postoperative delayed extubation (41.0% vs 26.4%), reflecting an approximately 1.5-fold higher risk.

**Table 5:** Postoperative recovery indicators among adult surgical patients.

Variable	Overall	Case group	Control group	*P*-value
No. of patients	840	212	628	
Ventilator-off time (min)	43.6 ± 27.46	45.3 ± 27.19	43.0 ± 27.55	.298
Extubation time (min)*	43.3 ± 25.90	59.0 ± 27.51	48.5 ± 28.98	<.001
Recovery time (min)	110.1 ± 36.85	117.4 ± 36.02	107.6 ± 36.84	.001
Delayed extubation				<.001
Yes	253 (30.1)	87 (41.0)	166 (26.4)	
No	587 (69.9)	125 (59.0)	462 (73.6)	

Data were presented as n (%) or mean ± SD.

SD = Standard deviation.

* Missing data.

**Figure 4.**
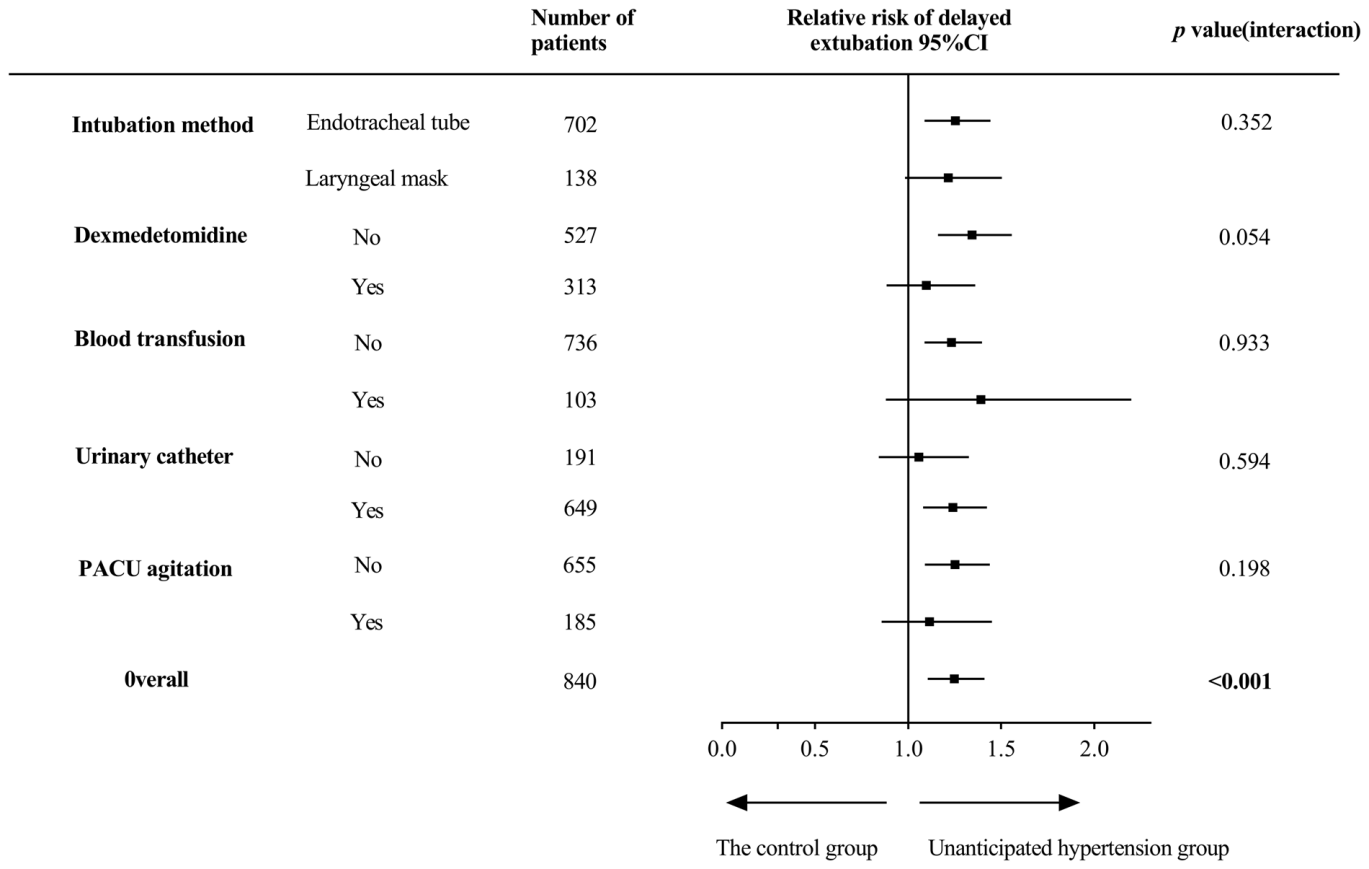
Short-term effects of unanticipated hypertension in emergence on delayed extubation. Forest plot of relative risk for intubation method, dexmedetomidine, blood transfusion, urinary catheter, and postoperative agitation subgroups. 95% CI = 95% confidence interval, PACU = postanesthesia care unit.

A sensitivity analysis was performed to evaluate the interaction between unanticipated hypertension and delayed extubation in elderly patients. As illustrated in Figure 4, subgroup analyses stratified by extubation method, intraoperative blood transfusion, use of dexmedetomidine, presence of a urinary catheter, and occurrence of EA in the PACU consistently demonstrated a similar magnitude of effect. The association between unanticipated hypertension and delayed extubation was robust across diverse patient subgroups.

## 4. Discussion

To date, numerous studies have investigated risk factors for acute postoperative hypertension, reporting an incidence range of 22.5% to 50.3%.^[10–12]^ In the present study, 212 patients experienced unanticipated hypertension, yielding an incidence of 25.2%. Although findings from prior investigations provide valuable reference, the distinct pathophysiological and clinical profiles of elderly surgical patients suggest that these factors may not be directly or fully applicable. With the growing aging population, the number of elderly patients undergoing surgical procedures has steadily increased. This population is particularly susceptible to perioperative hemodynamic instability owing to the high prevalence of comorbidities, age-related organ functional decline, and marked interindividual variability in drug responses.^[17]^ Given these unique physiological and clinical characteristics, elderly surgical patients warrant heightened attention from anesthesiologists. This study aimed to identify potential risk factors for unanticipated hypertension during the emergence and recovery phase in elderly surgical patients, which may inform optimized perioperative hemodynamic management and ultimately improve postoperative outcomes.

Our research was comparable to a US study that found that elderly patients are more vulnerable to drastic changes in blood pressure and are at higher risk of adverse events during emergence,^[18]^ which is consistent with our findings. Elderly surgical patients, as a high-risk group, frequently present with multiple comorbidities such as hypertension, diabetes, and chronic kidney disease. This study revealed that for every 10-year increment in age, the risk of unanticipated hypertension during anesthesia recovery increases by 2.02-fold. With advancing age, progressive declines in organ functional reserve, impaired sympathetic nervous system regulation, and reduced vascular elasticity^[19]^ collectively contribute to compromised circulatory control, thereby predisposing patients to significant perioperative hemodynamic instability.

In addition, our findings demonstrate that elderly female patients are more susceptible to unanticipated hypertension during emergence from anesthesia than their male counterparts. This sex-specific vulnerability may be attributed to several interrelated mechanisms: postmenopausal estrogen decline accelerates endothelial dysfunction and vascular stiffening, leading to impaired autonomic regulation;^[20]^ heightened sympathetic nervous system reactivity combined with reduced vagal tone in aging women predisposes to exaggerated hemodynamic responses.^[21]^ Furthermore, a 2020 study by Franconi et al^[22]^ reported sex-based differences in the pharmacokinetics of anesthetic agents – such as prolonged opioid clearance in women – which may disrupt autonomic balance and compromise circulatory control.

Multivariate regression analysis revealed that elderly patients experiencing intraoperative hypertension were 1.59 times more likely to develop unanticipated hypertension during emergence from general anesthesia compared with the control group. Intraoperative elevation in blood pressure can exacerbate hemodynamic instability through multiple pathophysiological mechanisms. Evidence indicates that fluctuations in intraoperative blood pressure may activate the renin-angiotensin-aldosterone system, thereby increasing cardiac afterload and impairing perfusion of vital organs.^[23]^ In our study, the incidence of intraoperative hypertension was 40.1% and 31.2% in the respective groups. Notably, no significant differences were observed between groups regarding preoperative history of hypertension or intraoperative use of vasoactive agents, suggesting adequate control of potential confounding factors. Consistent with our findings, Salmasi et al reported that patients with sustained intraoperative SBP exceeding baseline by ≥20% faced a 3.2-fold higher risk of cardiovascular adverse events in the PACU.^[24]^ This phenomenon may be attributed to compensatory suppression of sympathetic activity and dysregulation of peripheral vascular tone following acute hypertensive episodes.^[25]^ Further supporting this association, a cohort study of 1852 noncardiac surgical patients confirmed that marked intraoperative blood pressure variability is an independent predictor of vasoactive drug requirement in the PACU.^[26]^ These findings collectively align with our results and underscore the clinical relevance of intraoperative hemodynamic control. Importantly, approximately 76% of intraoperative hypertensive events may be preventable through precise monitoring of anesthetic depth and prophylactic administration of α/β-adrenergic receptor blockers.^[27]^ These data support the notion that optimized intraoperative blood pressure management represents a modifiable intervention target for improving postoperative outcomes in elderly surgical patients.

Our findings indicate that inadequate preoperative adherence to antihypertensive therapy and postoperative urinary catheter irritation represent 2 modifiable risk factors for unanticipated hypertension in elderly patients during emergence. A systematic review demonstrated that elderly surgical patients who miss ≥30% of prescribed antihypertensive doses preoperatively face a 2.3-fold higher risk of SBP exceeding 160 mm Hg upon emergence from anesthesia,^[28]^ likely due to subtherapeutic drug trough levels leading to rebound hypertension. This is further supported by a 2019 randomized controlled trial showing that patients with irregular β-blocker use had a significantly higher incidence of severe hypertension (≥180/110 mm Hg) at tracheal extubation compared to those with consistent medication use (28.1% vs 9.7%).^[29]^ These data underscore the importance of enhancing preoperative medication adherence assessment and management to optimize hemodynamic stability during the anesthesia recovery phase. Moreover, mechanical stimulation of the bladder trigone from indwelling urinary catheters can activate the sympathetic-adrenal medullary axis, resulting in a rapid rise in plasma norepinephrine concentration within 30 minutes of catheter insertion. A prospective cohort study reported that catheter-related discomfort induced acute hypertensive episodes (ΔSBP > 40 mm Hg) in 19.4% of elderly patients in the PACU, with 74% of such cases promptly resolved by administration of α1-adrenergic receptor blockers.^[30]^ Collectively, our results highlight that structured evaluation of urinary catheter necessity should be integrated into enhanced recovery after surgery protocols, as it effectively interrupts iatrogenic sympathetic overactivation and provides evidence-based support for achieving stable postoperative hemodynamics.

A total of 49 elderly patients experienced moderate to severe pain in the PACU, necessitating the administration of supplementary analgesics. The OR for postoperative pain was 5.692, markedly higher than any other risk factor, indicating that postoperative pain is the most significant predictor of unanticipated hypertension during emergence from general anesthesia. This finding aligns with prior evidence demonstrating that acute postoperative pain activates the sympathetic nervous system, resulting in elevated plasma norepinephrine levels and a consequent sharp increase in blood pressure.^[31]^ Furthermore, pain-induced activation of the hypothalamic-pituitary-adrenal axis enhances cortisol secretion, which potentiates vascular responsiveness to catecholamines.^[32]^ These 2 pathways synergistically contribute to pain-mediated blood pressure elevation, thereby adversely affecting recovery outcomes. Notably, this study also identified orthopedic surgery (OR = 2.01) and delayed initiation of patient-controlled analgesia (OR = 2.77) as independent risk factors for perioperative hypertension. The heightened nociceptive input in orthopedic procedures – attributable to extensive injury to skeletal muscle and periosteum – triggers the release of inflammatory mediators such as substance P and bradykinin, amplifying peripheral and central pain signaling and contributing to severe acute postoperative pain.^[33]^ Additionally, a 2022 randomized clinical trial^[34]^ demonstrated that inadequate early pain control may precipitate central sensitization, characterized by reduced excitability thresholds in spinal dorsal horn neurons; even subsequent analgesic administration may fail to normalize hemodynamic responses due to this “window of vulnerability” in blood pressure regulation. Collectively, postoperative pain likely serves as a central mediating factor, linking surgical stress to unanticipated hypertension through multiple interconnected mechanisms – including sympathetic-adrenal activation, neuroinflammatory signaling, and central sensitization. Therefore, optimizing perioperative analgesic strategies – such as implementing preoperative regional analgesia combined with immediate postoperative initiation of analgesic pumps – may represent a critical intervention to improve cardiovascular stability in elderly surgical patients.

Postoperative EA is a prevalent complication following general anesthesia, affecting 4.7% to 21.3% of adult patients. It is clinically characterized by disorientation, purposeless limb movements, and verbal agitation.^[35]^ Patients experiencing EA face a significantly elevated risk of adverse events, including tracheal tube displacement or increased mechanical stress on surgical wounds – complications that may precipitate postoperative hemorrhage or acute cardiovascular instability in severe cases.^[36]^ In the PACU, targeted sedation with intravenous propofol (0.5–1 mg/kg) has been established as an effective therapeutic strategy for managing EA. In this study, postoperative agitation was identified as an independent risk factor for unanticipated hypertension during emergence from general anesthesia in elderly patients (OR = 1.88, 95% CI: 1.26–2.78). Furthermore, the amplitude of SBP fluctuations in patients with agitation was 28.6% greater than that in the control group (*P* < .01). These findings are consistent with multiple prior studies demonstrating a strong association between EA and perioperative blood pressure elevation. Potential mechanisms underlying hemodynamic instability include excessive activation of the sympathetic-adrenal axis, leading to increased peripheral vascular resistance,^[37]^ and the establishment of a pain-irritability feedback loop.^[38]^ Collectively, these results indicate that postoperative agitation contributes to marked blood pressure variability in elderly surgical patients through interconnected neuroendocrine and sensory pathways. Therefore, implementing individualized sedation monitoring – such as using processed electroencephalographic indices to guide sedative dosing – may play a crucial role in preserving hemodynamic stability during the critical recovery phase.

In the sensitivity analysis, we evaluated the short-term postoperative recovery outcomes between the 2 patient groups. The results revealed that elderly patients who developed unanticipated hypertension during the emergence phase of anesthesia had a significantly prolonged length of stay in the PACU and were at higher risk for delayed postoperative extubation. Importantly, this association remained consistent across all predefined subgroups, indicating the robustness of the effect regardless of baseline patient characteristics. These findings imply that unanticipated hypertension episodes during the recovery period of elderly surgical patients may not only increase the clinical workload for healthcare providers but also impair operating room turnover rates and hinder the efficient utilization of critical PACU resources. Our study has some limitations. Stratification analysis of the severity and duration of blood pressure during emergence was not performed. Second, the retrospective cohort design limits the ability to capture detailed outcomes, particularly regarding postoperative cardiovascular and cerebrovascular complications, due to reliance on existing records. As a result, the long-term effects of unanticipated hypertension in elderly patients in the PACU remain to be fully identified and should be addressed in future prospective studies with more comprehensive follow-up.

In conclusion, our findings demonstrate that advanced age, female sex, absence of preoperative antihypertensive medication, orthopedic surgery, intraoperative hypertension, urinary catheterization, postoperative pain, and postoperative agitation were independently associated with an increased risk of unanticipated hypertension during emergence from anesthesia in elderly surgical patients. These results highlight the importance of early identification of high-risk individuals and support the implementation of individualized perioperative blood pressure management strategies to reduce the incidence of unanticipated hypertension and improve the perioperative prognosis of elderly patients.

## Acknowledgments

The authors thank AiMi Academic Services (www.aimieditor.com) for English language editing and review services.

## Author Contributions

**Conceptualization:** Li Xiang Yu.

**Funding acquisition:** Li Xiang Yu.

**Methodology:** Shuang Shuang Jiao.

**Data curation:** Shuang Shuang Jiao, Xing Jie Guo.

**Formal analysis:** Shuang Shuang Jiao, Xing Jie Guo.

**Visualization:** Li Xiang Yu.

**Project administration:** Shuang Shuang Jiao, Li Xiang Yu.

**Resources:** Shuang Shuang Jiao.

**Software:** Shuang Shuang Jiao, Xing Jie Guo.

**Investigation:** Xing Jie Guo.

**Supervision:** Xing Jie Guo, Li Xiang Yu.

**Validation:** Xing Jie Guo, Li Xiang Yu.

**Writing – original draft:** Shuang Shuang Jiao, Xing Jie Guo.

**Writing – review & editing:** Li Xiang Yu.
